# Partial Substitution of Alfalfa Hay by Stevia (*Stevia rebaudiana*) Hay Can Improve Lactation Performance, Rumen Fermentation, and Nitrogen Utilization of Dairy Cows

**DOI:** 10.3389/fvets.2022.899148

**Published:** 2022-05-18

**Authors:** Maocheng Jiang, Osmond Datsomor, Zhiqiang Cheng, Zitong Meng, Kang Zhan, Tianyu Yang, Yinghao Huang, Qi Yan, Guoqi Zhao

**Affiliations:** ^1^Institute of Animal Culture Collection and Application, College of Animal Science and Technology, Yangzhou University, Yangzhou, China; ^2^Institutes of Agricultural Science and Technology Development, Yangzhou University, Yangzhou, China; ^3^Joint International Research Laboratory of Agriculture and Agri-Product Safety, the Ministry of Education of China, Yangzhou University, Yangzhou, China

**Keywords:** stevia, lactation performance, nitrogen utilization, rumen fermentation, partial substitution

## Abstract

The objective of this study was to determine the effect of replacing isonitrogenous and isoenergetic basis alfalfa hay (AH) with stevia (*Stevia rebaudiana*) hay in dairy cow diets on nutrient digestion, milk performance, rumen fermentation, and nitrogen (N) utilization. In this study, 24 healthy Holstein lactating dairy cattle with a similar milk yield of 33.70 ± 2.75 (mean ± SD) kg, days in milk 95.98 ± 23.59 (mean ± SD) days, and body weight 587.75 ± 66.97 (mean ± SD) kg were selected and randomly allocated into three groups. The constituents of the three treatments were (1) 30.0% AH, and 0% stevia hay (SH) for the AH group; (2) 24.0% AH, and 6% SH for the 6% SH group; (3) 18.0% AH, and 12% SH for the 12% SH group. The substitution of AH with SH did not affect dry matter intake (DMI), gross energy (GE), and other nutrients intake but increased the digestibility of neutral detergent fiber (NDF) and acid detergent fiber (ADF). Compared with the AH diet, the cows fed the 6% SH diet had a higher milk yield and concentration of milk fat. Fecal and urinary nitrogen (N) were lower in cows fed a 6% SH diet than in cows fed the AH diet. Milk N secretion and milk N as a percentage of N intake were higher in cows fed a 6% SH diet than in cows fed AH diets. The concentration of ruminal volatile fatty acids, acetic acid, and ammonia-N were higher in cows fed a 6% SH diet than in cows fed an AH diet. By comparison, the 12% SH group did not affect milk yield, milk composition, N utilization, and rumen fermentation compared with the AH and 6% SH groups. In conclusion, it appears that feeding 6% SH, replacing a portion of AH, may improve lactation performance and N utilization for lactating dairy cows.

## Introduction

With the current surge in global forage crop prices, long-term dependence on alfalfa hay (AH) imports cannot sustain the rapid development of the dairy industry in southern China. Thus, there is an urgent need to investigate alternative, sustainable growing forage crops to promote the sustainable development of dairy husbandry in southern China. Through the investigation of medicinal plant resources, stevia is known to grow in many provinces in southern China, and the annual output is also considerable. In recent years, the development and utilization of stevia have mainly focused on its research as a sweetener, such as stevioside (1, 2). However, there are few reports on stevia in animal feeding experiments (3), and there are no reports on ruminants.

Stevia is a perennial herb native to the subtropical regions of South America (4). In China, stevia cultivation first began in the 1970s using varieties introduced from Japan (5). At present, it is planted in most parts of southern China, where it grows easily like any other vegetable crop even in the garden (6). In recent years, China has become one of the major stevia planting countries in the world, accounting for about 60–80% of the world's total production (6). Therefore, research on the development and utilization of stevia is also increasing. It is a kind of plant with remarkable physiological activity that has shown great value in the development and utilization of medicinal plant resources. Previous studies have found that stevia has the functions of regulating blood pressure (7, 8), lowering blood lipids (9), lowering blood sugar (10), anti-inflammatory and bactericidal (11), and antioxidant (12). Both earlier and current studies not only confirm the safety of stevia products but also find more and more benefits from its consumption for human health (13, 14). Stevia is rich in amino acids and protein (13). Wang et al. (15) added stevioside to piglet feed, both average feed intake and average daily gain increased throughout the experimental period. Geuns et al. (16) conducted a chicken intubation experiment, adding stevioside to the chicken feed at a dosage of 643–1,168 mg per chicken. Chicken excreta analysis found that most of the stevioside was not metabolized in the chicken, only about 2% was converted to steviol, and no stevioside and steviol were detected in the blood. At the same time, a long-term feeding experiment conducted on broilers showed that the feed intake and daily weight gain of broilers supplemented with 667 mg/kg stevioside tended to increase. In addition, broiler feeding experiments conducted by Atteh et al. (17) showed that the addition of stevioside significantly increased the daily gain of broilers. The reports of these animal experiments have shown that the beneficial chemicals contained in stevia have a positive effect on animal health.

Few previous studies have scientifically evaluated how stevia could be efficiently used in animal production, especially in dairy production. This experiment was conducted to investigate how the substitution of stevia hay (SH) for AH in diets affects lactation performance, nitrogen utilization, and rumen fermentation of dairy cows.

## Materials and Methods

All Holstein bovines used in this research were strictly cared for in accordance with the principles of the Institutional Animal Care and Use Committee (IACUC) of Yangzhou University (SYXK (Su) 2016-0019).

### Animals, Experimental Design, and Diets

The stevia planted and subsequently utilized in this study did not replace any crops on the farm, as it was the main cash crop grown on a field located at 32°51′N, 120°19′W, and 4 m above sea level. Stems with leaves of stevia were collected at a height of 15–20 cm above ground during the full-bud growth stage. After harvest, the stevia portion (stem and leaves) was naturally dried to achieve a moisture content of <10% (on a dry matter basis). SH was prepared by chopping the stevia stem with leaves to a length of 3–4 cm using a manual forage chopper. The experiment was conducted on the ruminant experimental farm of Yangzhou University (Yangzhou city, Jiangsu province, China). Twenty-four healthy Holstein lactating dairy cows with similar milk yields of 33.70 ± 2.75 (mean ± SD) kg, days in milk 95.98 ± 23.59 (mean ± SD) days, and body weight 587.75 ± 66.97 (mean ± SD) kg were selected and randomly allocated into three groups. The constituents of the three treatments were (1) 30.0% AH and 0% SH for the AH group (*n* = 8); (2) 24.0% AH and 6% SH for the 6% SH group (*n* = 8); (3) 18.0% AH and 12% SH for the 12% SH group (*n* = 8). At the start of the adaptation period, the pre-experimental diet was replaced by the experimental diet. The experiment lasted for 70 d, including a 10 d period for adaptation. All diets were isonitrogenous, and isoenergetic and met the nutritional requirements of the National Research Council NRC (Table 1). Cows were housed in individual stall barns with free access to drinking water, fed 3 times a day (07:00, 13:00, and 20:00 h) at 105% *ad libitum* intake, and were milked 3 times a day at 08:00, 14:30, and 21:00 h.

**Table 1:** Ingredients and nutrient composition of diets.

**Item**	**Teatment** ^**1**^		
	**AH**	**6% SH**	**12% SH**
**Ingredient, % of DM**
Alfalfa hay	30.0	24.0	18.0
Stevia hay	0.0	6.0	12.0
Corn silage	30.9	30.9	30.9
Ground corn	12.8	12.8	12.8
Soybean meal	13.7	13.7	13.7
Cottonseed meal	5.3	5.3	5.3
DDGS^2^	4.8	4.8	4.8
Slat	0.3	0.3	0.3
Premix^3^	2.2	2.2	2.2
**Composition, % of DM**
DM	52.7	52.7	52.7
OM^4^	88.9	88.6	88.2
CP	18.6	18.6	18.5
NDF	31.6	32.4	33.2
NFC^5^	37.9	36.9	35.7
ADF	21.7	22.3	22.9
EE	3.63	3.70	3.77
Starch	22.8	22.6	22.5
NE_L_,^6^ Mcal/kg	1.58	1.54	1.56

^1^*Teatments (DM basis): AH = 28.0% alfalfa hay (AH) and 0% Stevia hay (SH), 10% SH = 20.0% alfalfa hay (AH) and 10% Stevia hay (SH), and 18% SH = 12.0% alfalfa hay (AH) and 18% Stevia hay (SH)*.

*^2^DDGS = distillers dried grains with solubles.*

*^3^Premix was formulated to provide vitamin A, vitamin D3, vitamin E, manganese sulfate, zinc sulfate, magnesium oxide, limestone, and sodium chloride. Chemical composition of premix contained (DM basis) 2.6% Zn, 13.8% Ca, 11.7% Na, 17.4% Cl, 4.8% Mg, 0.4% S, 0.1% K, 800 mg/kg Mn, 11,000 IU of Vitamin A; 54,500 IU of Vitamin D, and 1,000 IU of Vitamin E.*

*^4^Organic matter (OM) = 100 - ash.*

*^5^Non-fibrous carbohydrate (NFC) calculated as NFC (%) = 100 - (% ash + % NDF + % CP + % EE). EE = ether extract.*

*^6^Predicted values from the National Research Council (NRC) (2001) model (18)*.

### Sample Collection and Analyses

The daily feed offered, orts, and spillages were collected and weighed to determine dry matter intake (DMI). The offered and refused feed amounts were recorded on the 3rd and 6th days every other week throughout the entire experimental period. All samples were dried in an oven at 65°C for 48 h, ground using a Wiley mill (1188Y, Thomaswiley, USA) with a 2-mm screen size, and then stored for further analyses. Feed was milled and analyzed for dry matter (DM), ash, crude protein (CP), neutral detergent fiber (NDF), acid detergent fiber (ADF), total nitrogen (N), starch, gross energy (GE), and ether extract (EE).

A 7-day digestion and metabolism test was conducted at the end of the formal period, in which the first 2 days were the adaptation period, and the last 5 days were sampled for data. Total collection of feces and urine was performed as described in Cherif et al. (18). Feces were dried and ground using a pulverizer (WF - 20B, Zhenfeng, Jiangsu, China,) for subsequent determination of DM, NDF, ADF, total N, starch, GE, ash, and organic matter (OM) content. For both total mixed ration (TMR) and fecal samples, DM was detected using AOAC method 930.15; CP was tested using the method described by Kjeldahl, with an azotometer (Scino KT260, FOSS, Hillerod, Denmark); NDF and ADF were measured using the methods described by Van Soest using a Fiber Analyzer (2000i, Ankom, New York, USA); total N were measured using the methods as described in Santana et al. (19), starch content was measured enzymatically (Megazyme, K-TSTA, Ireland), and ash content was measured according to AOAC method 942.05 (20).

Milk production was recorded every day and a 50 ml sample from the collected 3 consecutive milkings (6:00, 13:00, and 20:00 h) during weeks 1, 4, 8, and 12. Each sample was placed in tubes with a preservative (0.05%, benzoic acid) and submitted to Dairy One Cooperative Inc. (Shanghai, China) for analysis of protein, fat, lactose, and total solids (TS) and somatic cell count (SCC). Milk urea nitrogen (MUN) levels were measured with a Beckman BUN Analyzer 2 (Beckman Instruments Inc., Fullerton, CA). The energy corrected milk (ECM) value was calculated using the formula of Orth (21): ECM = (0.3246 × kg of milk) + (13.86 × kg of milk fat) + (7.04 × kg of milk protein). Feed efficiency = Milk/DMI.

The total collection of ruminal fluid samples (approximately 200 ml) was performed as described by Wang et al. (22). Briefly, whole-rumen content samples collected from 4 locations in the rumen were filtered through 2 layers of cheesecloth. The first 100–200 ml of fluid were discarded to reduce the risk of saliva contaminating the rumen fluid sample. Next, pH, volatile fatty acids (VFA), and ammonia nitrogen (NH_4_-N) of the sampled rumen fluid were detected. Following that, the pH (PB-21, Beijing Sartorius Scientific Instrument Co., Ltd) value was determined immediately afterward the samples were stored at −20°C for the determination of VFA and NH_4_-N. VFA was determined by gas chromatography (Thermo Fisher Scientific, Waltham, MA, USA), with the detection method based on the previous research of Wu et al. (23). NH_4_-N was detected using the method described by Lamminen et al. (24).

### Statistical Analysis

Data are presented as the means and the standard error of the mean (SEM). Significant differences were determined by one-way ANOVA and Tukey's multiple comparison tests. The analysis was conducted using the SPSS Statistics software, version 20.0 (IBM Corp., Armonk, NY, USA) (25). Additionally, a *T*-test was applied to analyze the chemical composition data of hay. Statistical significance was defined at *p* < 0.05 with highly significant values at *p* < 0.01; trends were declared at 0.05 < *p* ≤ 0.1.

## Results

### AH and SH Composition

Alfalfa hay in the diet was partially replaced by SH based on isonitrogenous and isoenergetic. The composition of TMR and hay ingredients is shown in Tables 1, 2. As shown in Table 1, compared with the control group, AH was replaced by SH by 6% and 12% in the treatment group. As can be seen from Table 2, CP and non-fibrous carbohydrate (NFC) content were 8.2 and 44.3% lower for SH than for AH (*p* < 0.01). EE, NDF, ADF, and ash content were 65.1, 54.9, 35.0, and 14.6% units higher in SH than AH (*p* < 0.01).

**Table 2:** Chemical composition of alfalfa hay (AH) and stevia hay (SH) used in the diets (*n* = 7).

**Item, % of DM**	**Type of hay**		**SEM**	***P*-value**
	**Alfalfa**	**Stevia**		
DM	90.88	92.30	0.41	0.16
CP	16.21	14.88	0.33	<0.01
EE	1.89	3.12	0.24	<0.01
NDF	32.7	50.65	0.44	<0.01
ADF	29.54	39.88	0.46	<0.01
Ash	11.42	13.09	0.40	<0.01
NFC	32.77	18.25	0.64	<0.01

*NFC, non-fibrous carbohydrate*.

### Intake and Digestibility

The total amount of nutrient intake and apparent total-tract nutrient digestibility of the three treatment diets are shown in Table 3. Intake of DM, OM, CP, NDF, ADF, GE, and starch was similar between the experimental diets as presented in Table 3. Apparent total-tract digestibility of DM, OM, and CP was not significant among the three treatment diets. Besides, apparent total-tract digestibility of NDF, ADF, and GE was significantly higher in the 6% SH and 12% SH group than the AH group (*p* < 0.01).

**Table 3:** Effect of partial substitution of AH by SH on dry matter intake (DMI) and digestibility.

**Item**	**Treatment**			**SEM**	***P*-value**
	**AH**	**6% SH**	**12% SH**		
**Intake, kg/d**
DM	23.40	24.65	25.07	0.80	0.69
OM	21.44	22.70	22.96	0.52	0.47
CP	4.32	4.43	4.54	0.24	0.94
NDF	7.35	7.96	8.16	0.31	0.57
ADF	5.01	5.48	5.53	0.27	0.71
Starch	5.01	5.34	5.50	0.22	0.68
Gross energy (Mcal/d)	120.25	121.13	122.63	0.79	0.48
**Digestibility, %**
DM	66.05	67.58	66.36	0.71	0.67
OM	67.59	68.79	69.21	0.29	0.05
CP	66.65	67.55	67.33	0.26	0.38
NDF	38.99^b^	41.38^a^	40.66^a^	0.33	<0.01
ADF	32.32^b^	35.51^a^	36.19^a^	0.45	<0.01
Gross energy	69.00	70.88	70.75	0.51	0.25

^a, b^*Means within a row with different superscripts differ (p <0.05)*.

### Milk Production, Milk Composition, and Feed Efficiencies

The milk production, milk composition, and feed efficiencies of the three treatment diets are presented in Table 4. Compared with AH, the yield of milk was significantly increased for cows fed in the 6% SH group (*p* = 0.033). Fat yield in the 6% SH group was significantly higher than that in the AH group (*p* = 0.02). However, the two treatment groups had no significant effect on milk composition compared with the control group. Similarly, feed efficiency was similar between cows fed the three treatment diets.

**Table 4:** Effect of partial substitution of AH by SH on milk yield and milk composition.

**Item**	**Treatment**			**SEM**	***P*-value**
	**AH**	**6% SH**	**12% SH**		
**Yield, kg/d**
Milk	34.03	35.80	35.51	0.34	0.07
Fat	1.35^b^	1.53^a^	1.46^ab^	0.03	0.02
Protein	1.20	1.27	1.16	0.02	0.12
Lactose	1.57	1.69	1.66	0.03	0.30
ECM	37.37	39.84	38.42	0.54	0.18
Feed efficiency	1.47	1.49	1.47	0.05	0.98
**Composition, %**
Fat	3.79	3.89	3.81	0.07	0.85
Protein	3.52	3.54	3.27	0.06	0.14
Lactose	4.62	4.73	4.67	0.07	0.79
MUN, mg/dL	12.14	12.76	12.03	0.18	0.22

^a, b^*Means within a row with different superscripts differ (p <0.05)*.

### Nitrogen Utilization

As experimental diets were isonitrogenous, dietary inclusion of SH (6 or 12%) had no effect on N intake between treatments and averaged 660.21 g/days (Table 5). There was a significantly increased in the N content of milk from the 6% SH group compared with the AH group (*p* < 0.01). The retained N content was significantly higher in the SH group than the AH group (*p* = 0.02). However, the 6% SH group was significantly reduced fecal N excretion compared with the AH group (*p* = 0.022). Similarly, the urinary N secretion was significantly higher for cows fed the AH diet than for those fed the 6% SH treatment diets (*p* < 0.01).

**Table 5:** Effect of partial substitution of AH by SH on nitrogen utilization.

**Item**	**Treatment**			**SEM**	***P*-value**
	**AH**	**6% SH**	**12% SH**		
N intake, g/d	657.75	660.38	662.50	4.54	0.92
Milk *N* secretion, g/d	194.25^b^	217.88^a^	207.87^ab^	3.28	<0.01
% of *N* intake	29.57^b^	33.02^a^	31.40^ab^	0.52	0.02
Fecal *N* secretion, g/d	211.88	188.88	196.13	4.15	0.06
% of *N* intake	32.26^a^	28.57^b^	29.58^ab^	0.61	0.03
Urinary *N* secretion, g/d	208.38^a^	189.00^b^	199.01^ab^	3.10	0.03
% of *N* intake	31.65^a^	28.60^b^	30.05^b^	0.40	<0.01
Retained *N*, g/d	43.25^b^	64.75^a^	59.50^a^	3.49	0.02
% of *N* intake	6.52^b^	9.81^a^	8.97^a^	0.52	0.02

^a, b^*Means within a row with different superscripts differ (p <0.05)*.

### Rumen Fermentation

Rumen fermentation characteristics of the AH and SH groups are presented in Table 6. For ruminal NH_3_, the only change observed was between cows fed 6% SH and those fed the AH diet (*p* = 0.02), and there were no differences between the 6 and 12% SH diets (*p* = 0.21). Similarly, there was a significant increase in total VFA and acetic acid from the 6% SH group compared with the AH group (*p* = 0.028 and *p* = 0.021). There were no significant differences in rumen pH value (*p* = 0.37), molar proportion of propionic (*p* = 0.48), butyric acid (*p* = 0.74), and the ratio of acetic to propionic acid between the SH and AH diets (*p* = 0.18).

**Table 6:** Effect of partial substitution of alfalfa hay by stevia hay on ruminal pH, NH_3_, and volatile fatty acids (VFA).

**Item**	**Treatment**			**SEM**	***P*-value**
	**AH**	**6% SH**	**12% SH**		
pH	6.23	6.28	6.21	0.02	0.37
NH_3_, mg/dL	14.03	12.54	13.26	0.27	0.06
Total VFA, m*M*	89.77	96.67	92.13	1.29	0.08
**VFA proportion, m** ***M*** **/100 m** ***M***
Acetic acid	57.37	63.55	59.62	0.73	0.06
Propionic acid	17.81	18.29	18.66	0.28	0.48
Butyric acid	12.33	12.14	11.65	0.36	0.74
Acetic: propionic acid	3.24	3.48	3.20	0.07	0.18

## Discussion

### Chemical Composition of SH

*Stevia rebaudiana* is a perennial herb of significant economic value due to its high content of natural, dietetically valuable sweeteners in its leaves (26, 27). The nutrient composition of AH and SH was tested by our laboratory (Table 2). The stems and leaves of stevia plants contain 14.88% CP, 3.12% EE, 50.65% NDF, and 39.88% ADF. Several previous studies have shown that SH contains between 10 and 20.42% of CP and between 2.7 and 5.0% of EE (28–31). This is overall consistent with our results. Preliminary analysis based on present and past results showed that the changes in CP and EE contents are also affected by environment and regions. Comparing the nutritional components of SH with AH, it was found that SH had relatively good CP content and EE content. In addition, the NDF content of SH was higher than AH, with preferable digestibility and palatability. Therefore, SH was used to replace partial AH to explore whether SH can be used as a feed source for lactating dairy cows.

### Feed Intake, Digestibility, and Milk Production

In the present study, isonitrogenous and isoenergetic diets with similar chemical compositions between the SH and AH diets were fed (Table 1). The average CP and EE values of the three diets were 18.57 and 3.7%, respectively. Compared with the AH group, the average DMI of the 12% SH group increased by 7.14%. However, there was no effect on DMI by the cows fed the three diets. Thus, there was no significant difference in digestibility (OM, CP, NDF, ADF, starch, and GE) of the three treatment groups. Previous reports were only about the application of stevia extract (stevioside) in animal production. Following reports of Geuns et al. (16), the addition of stevia to diets had no significant effect on the DMI of broilers. In another study on weaning pigs, feed consumption was increased by 4.4% and weight gain by 3.1% (32). Generally, *stevia rebaudiana* is regarded as a natural sweetener, and sweeteners can promote animal feed intake (3). However, the result of the present experiment did not align with earlier feed intake results. This result may be due to the higher NDF of SH increasing satiety. Similarly, apparent total-tract digestibility of DM, CP, and GE averaged 66.66, 67.18, and 70.21%, respectively, and was not affected by dietary treatments. In contrast, the total-tract digestibility of OM, NDF, and ADF was higher for cows fed SH diets compared with cows fed an AH diet. Previous studies have shown that stevia can increase the abundance of beneficial bacteria and reduce the abundance of harmful bacteria in the gastrointestinal tract (33). Stevioside is one of the main chemical components of SH, accounting for 10% of the leaves. It may be forthis reason that the apparent digestibility of NDF and ADF in the SH group was higher than in the AH group.

For milk production, the yield of milk tended to be higher for cows fed the 6% SH group compared with the AH group. Despite the increased replacement ratio of stevia, the milk yield of the 12% SH group did not increase. Notably, milk fat content showed the same trend. Acetic acid is the main lipogenic precursor in the mammary gland during lactation has been reported (34). Therefore, the level of acetic acid which indicates cattle rumen fermentation could partially cause the variation in milk yield and fat. DMI and milk yield were not significantly affected when alfalfa was replaced with stevia in dairy cow diets. Similarly, feed efficiency was not affected.

### Nitrogen Metabolism

Previous studies have shown that the efficiency of converting nitrogen in dietary N of dairy cows into milk nitrogen is relatively low; most of the nitrogen from food is excreted through feces and urine, which also pollutes the environment (35). In the present experiment, when expressed as a proportion of N intake, the efficiency of N use for milk N secretion was higher for cows fed a 6% SH diet vs. cows fed an AH diet. It is well known that urea in the milk equilibrates rapidly with other body fluids, such as plasma (36), reflecting not only protein metabolism but also the inefficiency of N utilization (37). Previous studies have shown that milk N secretion is associated with protein degradation rate in the rumen (38). So it may also be due to the fact that the degradable protein of SH was higher than AH. Besides, more studies should be done with a wider range of dietary CP to determine the value that captures the dietary situations that maximize protein yield while minimizing urinary urea N. Nitrogen in feces is mainly related to undigested feed nitrogen and endogenous nitrogen (39). Overall, fecal N excretion and concentration in the AH group were higher than in the 6% SH group. This result also confirms that the protein degradation of SH was higher than AH. In addition, nitrogen retention may depend on the amount of digestible protein and the amino acid (AA) composition of the protein, as well as the energy available for the protein to accumulate in the body (40). In conclusion, the changes in these values confirm that the protein degradation rate of SH in the rumen was higher than AH in this study.

### Rumen Fermentation

Results of previous studies showed that a higher total concentration of ruminal VFA was indicative of higher nutrient digestibility (22). The higher ruminal total VFA concentration in cows fed the 6% SH diet was indicative of higher nutrient digestibility, which indirectly explains the increase in milk production. Excessive concentrations of NH_3_ in the rumen resulted in high levels of N excreted mainly in the urine (41). Consistent with expectations, the ruminal NH_3_ concentrations are well correlated with MUN and urinary N excretion. In this study, NH_3_ content in the 6% treatment group was significantly lower than that in the AH group. The same results were obtained from urine nitrogen secretion. Similarly, Benchaar et al. (42) reported that dietary treatments that increased ruminal NH_3_ concentration also increased urinary N excretion. Meanwhile, replacing AH with SH in dairy cow diets did not change the molar proportions of acetic and propionic acid. Although the concentration of acetic acid tended to change in the three diets, the ratio of acetic/propionic acid did not change. In short, using a certain proportion of SH instead of AH is conducive to the rumen fermentation of dairy cows, but it will not increase the proportion.

### Conclusions

Results of this study revealed that SH can partially substitute AH in the diet of dairy cows on an isonitrogenous and isoenergetic basis without adverse effects on intake. Cows fed the 6% SH diet demonstrated a higher yield of milk and milk fat content. Compared with the AH group, cows fed the 6% SH diet had higher nutrient digestibility and nitrogen utilization. However, the 12% SH group did not get the corresponding effect. Thus, these results suggest that feeding 6% SH, replacing a portion of AH, improves lactation performance and nitrogen utilization for lactating dairy cows.

## Data Availability Statement

The original contributions presented in the study are included in the article/supplementary material, further inquiries can be directed to the corresponding author.

## Ethics Statement

The animal study was reviewed and approved by accordance with the principles of the Institutional Animal Care and Use Committee (IACUC) of Yangzhou University (SYXK (Su) 2016-0019).

## Author Contributions

MJ and GZ designed the whole experiment and verified the validity of the experiment and checked the results. KZ, QY, and OD performed the experiment, including chemical analysis, and statistical analysis. MJ, TY, and ZM worked on the manuscript. ZC, YH, and ZM participated in the experiment design and gave valuable advice. All authors have read and approved the final version of this manuscript.

## Funding

This study was supported by the National Natural Science Foundation of China (No. 31972589), the China Agriculture Research System of MOF and MARA, and the Excellent Doctoral Dissertation Fund of Yangzhou University.

## Conflict of Interest

The authors declare that the research was conducted in the absence of any commercial or financial relationships that could be construed as a potential conflict of interest.

## Publisher's Note

All claims expressed in this article are solely those of the authors and do not necessarily represent those of their affiliated organizations, or those of the publisher, the editors and the reviewers. Any product that may be evaluated in this article, or claim that may be made by its manufacturer, is not guaranteed or endorsed by the publisher.
